# Predicting Flavin and Nicotinamide Adenine Dinucleotide-Binding Sites in Proteins Using the Fragment Transformation Method

**DOI:** 10.1155/2015/402536

**Published:** 2015-04-27

**Authors:** Chih-Hao Lu, Chin-Sheng Yu, Yu-Feng Lin, Jin-Yi Chen

**Affiliations:** ^1^Graduate Institute of Molecular Systems Biomedicine, China Medical University, Taichung 40402, Taiwan; ^2^Graduate Institute of Basic Medical Science, China Medical University, Taichung 40402, Taiwan; ^3^Department of Information Engineering and Computer Science, Feng Chia University, Taichung 40724, Taiwan; ^4^Master's Program in Biomedical Informatics and Biomedical Engineering, Feng Chia University, Taichung 40724, Taiwan; ^5^Institute of Bioinformatics and Systems Biology, National Chiao Tung University, Hsinchu 30068, Taiwan

## Abstract

We developed a computational method to identify NAD- and FAD-binding sites in proteins. First, we extracted from the Protein Data Bank structures of proteins that bind to at least one of these ligands. NAD-/FAD-binding residue templates were then constructed by identifying binding residues through the ligand-binding database BioLiP. The fragment transformation method was used to identify structures within query proteins that resembled the ligand-binding templates. By comparing residue types and their relative spatial positions, potential binding sites were identified and a ligand-binding potential for each residue was calculated. Setting the false positive rate at 5%, our method predicted NAD- and FAD-binding sites at true positive rates of 67.1% and 68.4%, respectively. Our method provides excellent results for identifying FAD- and NAD-binding sites in proteins, and the most important is that the requirement of conservation of residue types and local structures in the FAD- and NAD-binding sites can be verified.

## 1. Background

Over the past 12 years, projects involving structural genomics have generated structural data for ~12,000 proteins within the Protein Data Bank (PDB) [1]. For most of these proteins, however, biological function is unknown. It is therefore important to develop computational methodologies that can identify a protein's function from its structure. Many biochemical processes depend on interactions between proteins and cofactors, such as metal ions, vitamins, and adenine dinucleotides, for example, flavin adenine dinucleotide (FAD) and nicotinamide adenine dinucleotide (NAD). Adenine dinucleotides play important roles in many central biological processes, including DNA repair [2, 3], glycolysis, photosynthesis, and transcription [4–7]. By June 2010, 5293 proteins in PDB were annotated “nucleotide binding,” and nucleotides constitute ~15% of biologically relevant ligands [8]. These statistics demonstrate how ubiquitous and essential protein-nucleotide interactions are to biological processes.

Although protein-ligand interactions are fundamental to most biochemical reactions, structural information concerning these binding sites is still inadequate. Once ligand-binding sites can be predicted from structural data, putative functions can be assigned to these proteins. More complete annotation of protein function will benefit both basic science and the pharmaceutical industry. Mutations or deletions within these ligand-binding domains often alter biochemical reactions and are the root causes of many diseases. This makes binding sites attractive targets for drug therapies, including anticancer chemotherapy. In recent years computational methods have been used to identify ligand-binding sites within proteins. These methods include empirical approaches [9], support vector machines (SVM) [8, 10, 11], random forest [12, 13] and artificial neural networks [14], and structure comparison approaches [15–17]. These prediction methods can be divided into two broad categories: ones that use protein-sequence information, for example, amino acid composition, position-specific scoring matrix, and physicochemical properties, and ones that use protein-structure information, for example, dihedral angles, secondary structure, and 3D-structure comparison. The most effective prediction methodologies, however, tend to use a combination of sequence and structure data.

The structural genomics initiative resolves 20 new protein structures each week, and more than 60,000 structures have been deposited into PDB. The functional surfaces of proteins, which interact with cofactors, tend to be more structurally conserved than internal structures [18]. Residues that form a functional binding region are usually quite close to one another when the three-dimensional structure of a protein is examined. In addition, binding regions typically constitute only 10–30% of the entire protein [19–21]. We took advantage of previously generated structural information and used the fragment transformation method [22] to identify new binding sites for the NAD and FAD ligands.

## 2. Results

### 2.1. Residues that Bind NAD or FAD

**Figure 1:**
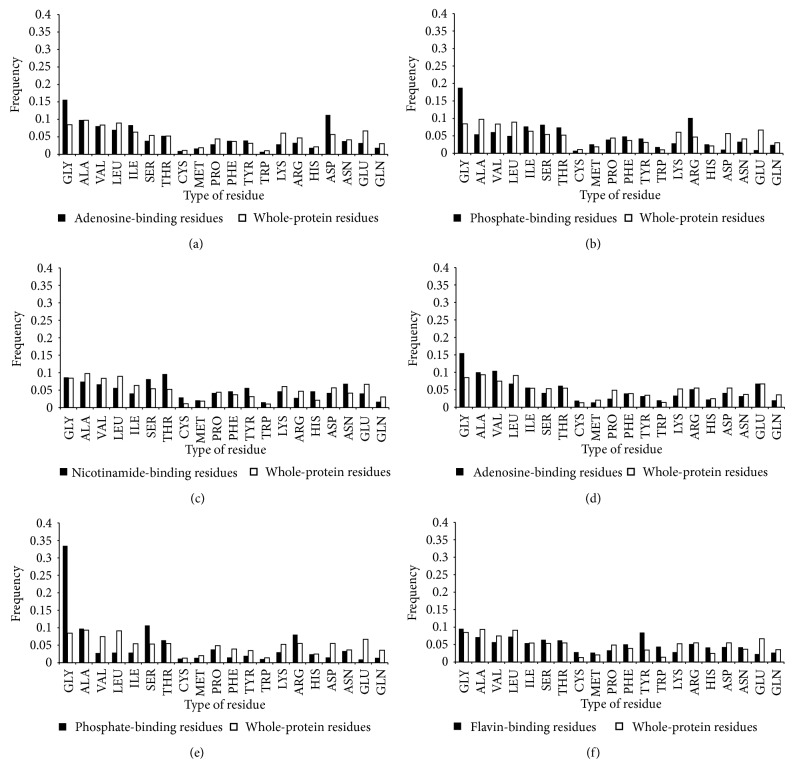
Amino acid frequencies within NAD-/FAD-binding sites. Frequencies within NAD-/FAD-binding sites (black) are compared with whole-protein frequencies (white). (a) Adenosine-binding of NAD. (b) Phosphate-binding of NAD. (c) Nicotinamide-binding of NAD. (d) Adenosine-binding of FAD. (e) Phosphate-binding of FAD. (f) Flavin-binding of FAD. The preferred types of amino acids surrounding the different moiety of NAD/FAD are shown.

To characterize the structural environment of NAD-/FAD-binding sites, we compared binding-site residues to whole-protein residues. The three-dimensional structure of the NAD/FAD molecule was divided into three moieties according to function. Within the spherical environment of NAD, the adenosine-binding site typically contained glycine, isoleucine, tyrosine, and aspartic acid residues; the phosphate-binding site contained glycine, isoleucine, serine, threonine, methionine, phenylalanine, tyrosine, tryptophan, arginine, and histidine residues; and the nicotinamide-binding site contained serine, threonine, cysteine, phenylalanine, asparagine, tyrosine, tryptophan, histidine, and asparagine residues. For FAD, adenosine was bound by glycine, valine, cysteine, and tryptophan; phosphate was bound by glycine, serine, and arginine; and flavin was bound by cysteine, methionine, phenylalanine, tyrosine, tryptophan, and histidine. The residue types whose ratio of binding-site residues frequency to whole-protein residues frequency was greater than 1.2 were listed above. As such, the binding residues were primarily polar residues, containing charged groups, amide groups, and nucleophilic groups (Figure 1).

**Figure 2:**
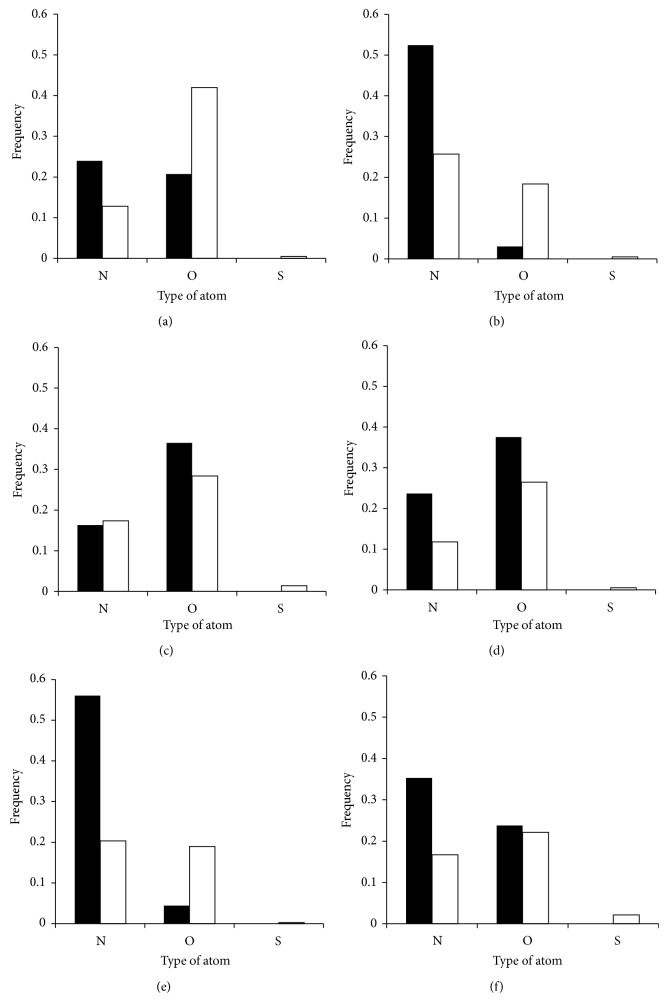
Atom-type frequencies within NAD-/FAD-binding sites. Frequencies for both backbone (black) and side-chain (white) atoms are shown. (a) Adenosine-binding of NAD. (b) Phosphate-binding of NAD. (c) Nicotinamide-binding of NAD. (d) Adenosine-binding of FAD. (e) Phosphate-binding of FAD. (f) Flavin-binding of FAD. The preferred types of atoms surrounding the different moiety of NAD/FAD are shown.

We also characterized the types of atoms that were within 3.5 Å of the three moieties of each NAD/FAD ligand (Figure 2). Nicotinamide and flavin moieties were most commonly associated with nitrogen and oxygen atoms within the backbone and side-chains of the protein. Phosphate moieties were commonly bound by backbone and side-chain nitrogen or side-chain oxygen. Each ligand moiety preferentially bound certain atoms within certain residues.

### 2.2. Prediction Performance

We chose two criteria to evaluate the performance of our binding-site predictions: performance at less than 5% FPR and the Matthews correlation coefficient (MCC). We used a combination of features that included the number of aligned residues, RMSD, BLOSUM, and DSSP. Using a 5% FPR threshold, NAD-binding sites were predicted with an accuracy of 93.46%, a sensitivity of 67.09%, and an MCC of 0.52. Under these same conditions, FAD-binding-site predictions yielded 93.59% accuracy, 68.43% sensitivity, and an MCC of 0.54 (Table 1). When MCCs were maximized, NAD-binding proteins were identified with 95.34% accuracy, 57.88% sensitivity, 97.64% specificity, and an MCC of 0.57. Under these same conditions, FAD-binding residues were identified with an accuracy of 94.33%, a sensitivity of 64.13%, a specificity of 96.27%, and an MCC of 0.55 (Table 2). These data indicated that our method could predict binding residues for these two ligands.

### 2.3. Comparison with Other Methods

We next compared our results with other prediction methodologies. For these comparisons we chose two published methods that use similar criteria for analyzing these kinds of ligand-protein complexes [10, 11]. These chosen methods assign binding or nonbinding status to each residue within NAD-/FAD-binding proteins. Because these published methods use an equal number of binding and nonbinding residues, we applied our prediction method to a similar dataset to make the results comparable. Random-selection processes were performed five times for all nonbinding residues within ligand-protein complexes to generate the same scale for binding and nonbinding residues within each protein. For NAD-binding proteins, our method predicted binding residues with a sensitivity of 86.21% and an MCC of 0.75 compared with 86.13% and 0.75 for the method developed by Ansari and Raghava [10] (Table 3). For FAD-binding proteins, our method yielded 85.68% sensitivity and an MCC of 0.75. These values compared with the performance of the published method (83.36% and 0.66) developed by Mishra and Raghava [11] (Table 4). Our method, therefore, has similar performance in NAD-binding sites predicted but better in FAD-binding sites. However, in native proteins, the number of binding and nonbinding residues should not be equal. The equal number model needs to be further discussed.

### 2.4. Template Matching

**Figure 3:**
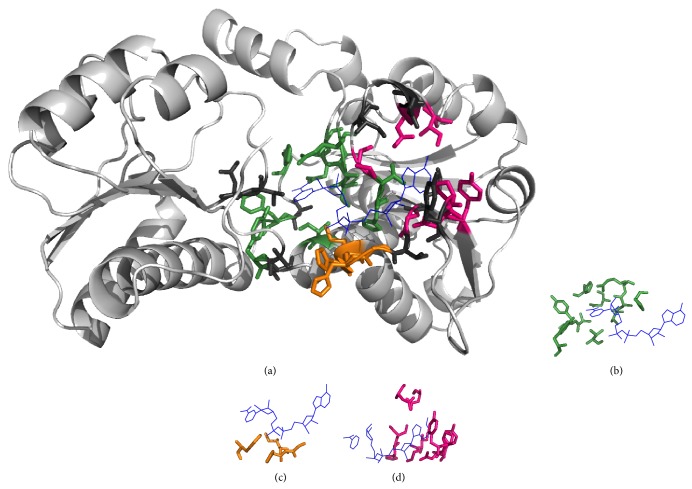
Identification of NAD-binding sites. (a) Chain A of D-2-hydroxyisocaproate dehydrogenase (PDB ID:1DXY) was the query protein. Templates were constructed from (b) D-Lactate dehydrogenase (chain A; PDB ID:3KB6), (c) phosphoglycerate dehydrogenase (chain A; PDB ID:1YBA), and (d) C-terminal-binding protein/brefeldin A-ADP ribosylated substrate (chain A; PDB ID:1HKU).

**Figure 4:**
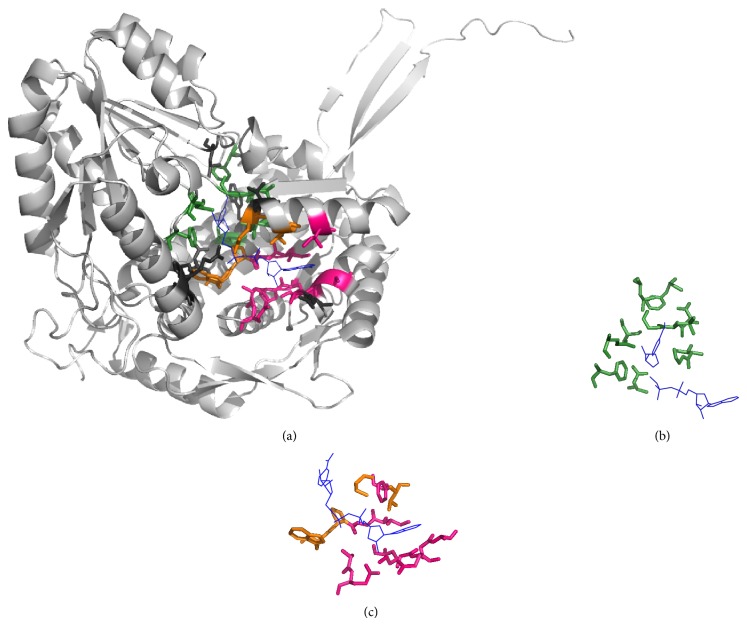
Identification of NAD-binding sites. (a) Chain C of 5-carboxymethyl-2-hydroxymuconate semialdehyde dehydrogenase (PDB ID:2D4E) was the query protein. Templates were constructed from (b) aldehyde dehydrogenase (chain A; PDB ID:3B4W) and (c) 1-pyrroline-5-carboxylate dehydrogenase (chain A; PDB ID:2EHU).

**Figure 5:**
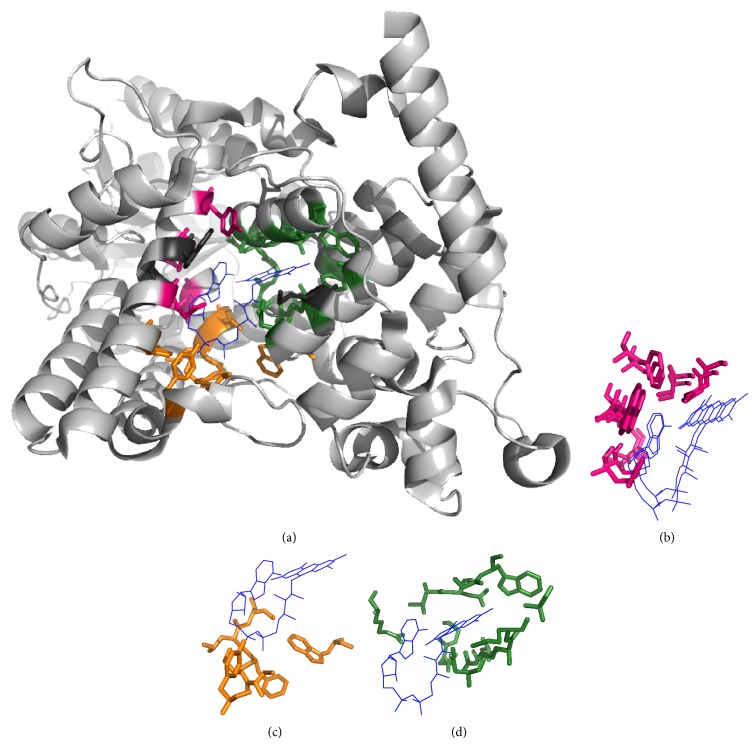
Identification of FAD-binding sites. (a) Chain A of deoxyribodipyrimidine photolyase (PDB ID:1OWL) was the query protein. Templates were constructed from (b) human cryptochrome DASH (chain X; PDB ID:2IJG), (c) photolyase-like domain of cryptochrome 1 (chain A; PDB ID:1U3C), and (d) photolyase (chain A; PDB ID:1IQR).

**Figure 6:**
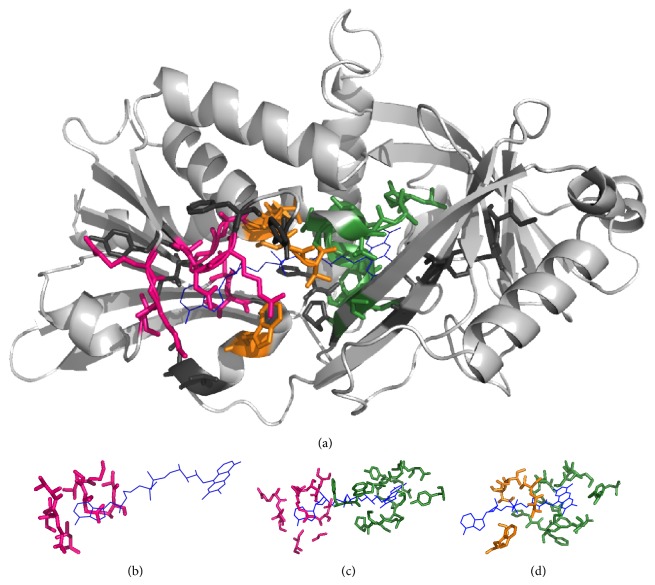
Identification of FAD-binding sites. (a) Chain H of D-amino acid oxidase (PDB ID:1DDO) was the query protein. Templates were constructed from (b) putidaredoxin reductase (chain B; PDB ID:1Q1R), (c) D-amino acid oxidase (chain A; PDB ID:1C0I), and (d) glycine oxidase (chain B; PDB ID:1NG3).

Figures 3–6 show alignments of predicted NAD-/FAD-binding proteins and corresponding templates. Structures within these figures were drawn using PyMOL [23] and color coded: light gray for the query protein; blue lines for the ligand; hot pink, orange, and forest sticks for adenosine-, phosphate-, and nicotinamide-/flavin-binding residues that are predicted correctly; and dark gray sticks for nonbinding residues that are predicted to be binding residues. Our method accurately identified 21 NAD-binding residues within chain A of D-2-hydroxyisocaproate dehydrogenase (PDB ID:1DXY) [24, 25], with ten false positives (Figure 3). Nine nicotinamide-binding residues were identified based on D-Lactate dehydrogenase (chain A; PDB ID:3KB6) [26, 27], three phosphate-binding residues were identified based on phosphoglycerate dehydrogenase (chain A; PDB ID:1YBA) [28], five adenosine-binding residues were identified based on C-terminal-binding protein/brefeldin A-ADP ribosylated substrate (chain A; PDB ID:1HKU) [29], and four were identified based on other protein templates. Our method also accurately predicted 23 NAD-binding residues within chain C of 5-carboxymethyl-2-hydroxymuconate semialdehyde dehydrogenase (PDB ID:2D4E), with only eight false positives (Figure 4). Nine nicotinamide-binding residues were identified based on aldehyde dehydrogenase (chain A; PDB ID:3B4W), three phosphate-binding and eight adenosine-binding residues were identified based on 1-pyrroline-5-carboxylate dehydrogenase (chain A; PDB ID:2EHU), and three were identified based on other protein templates.

For the FAD-binding proteins, our method accurately predicted chain A of deoxyribodipyrimidine photolyase (PDB ID:1OWL) [30] which contains 24 residues that bind FAD (Figure 5) and only six false positives occurred. Three adenosine-binding residues were identified based on human cryptochrome DASH (chain X; PDB ID:2IJG) [31, 32], six phosphate-binding residues were identified based on photolyase-like domain of cryptochrome 1 (chain A; PDB ID:1U3C) [33], eleven flavin-binding residues were identified based on photolyase (chain A; PDB ID:1IQR) [34], and four were identified based on other protein templates. In addition, 30 FAD-binding residues were accurately predicted within chain H of D-amino acid oxidase (PDB ID:1DDO) [35] with 14 false positives. Five adenosine-binding residues were predicted based on putidaredoxin reductase (chain B; PDB ID:1Q1R) [36, 37], three adenosine-binding and nine flavin-binding residues based on D-amino acid oxidase (chain A; PDB ID:1C0I) [38], three phosphate-binding and five flavin-binding residues based on glycine oxidase (chain B; PDB ID:1NG3) [39], and five based on other protein templates (Figure 6).

## 3. Discussion

Small molecular cofactors (ligands) are essential for cells to perform numerous biological functions. NAD and FAD, for example, bind to proteins that play critical roles in energy transfer, energy storage, and signal transduction, to name just a few. To understand the mechanism by which these ligands affect protein function, it is important to identify ligand-binding residues within relevant proteins. The experimental identification of these interacting residues is so difficult; however, that computational methods to accomplish this task are in high demand.

**Figure 7:**
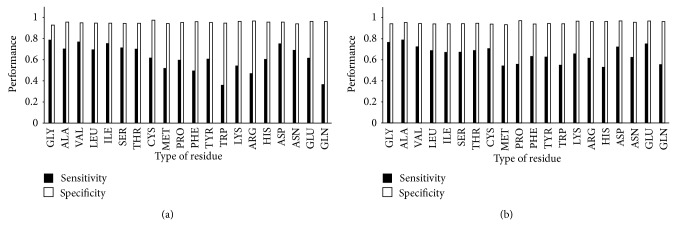
Sensitivity and specificity associated with each amino acid in NAD-/FAD-binding-site predictions. (a) NAD. (b) FAD.

Here we developed a structure comparison method that uses both sequence and structure information to predict NAD-/FAD-binding residues within proteins. This approach also provides valuable information concerning the microenvironment of the protein-ligand interaction. The composition of NAD-/FAD-binding residues that we identified here is generally similar to previous studies [10, 11]. Interestingly, glycine was the most frequent binding residue, binding to NAD through phosphate or adenosine moieties more often than through the nicotinamide moiety. In contrast, arginine preferentially interacted with phosphate moieties and aspartic acid preferentially interacted with adenosine moieties of NAD, whereas threonine, cysteine, and histidine bound to nicotinamide. The most common residue within FAD-binding sites was also glycine, which preferentially bound phosphate and adenosine moieties. Serine interacted with phosphate moieties, whereas cysteine, tyrosine, and tryptophan primarily bound to nicotinamide. By taking advantage of this kind of structural information, details concerning these critical binding sites may be revealed. To investigate the influence of amino acids on prediction performance, the sensitivity and specificity associated with each residue were calculated (Figure 7). For NAD-binding-site predictions, specificity for each residue was excellent (0.927–0.966), but sensitivity was relatively low for phenylalanine, tryptophan, arginine, and glutamine which were less than 0.5. For FAD-binding sites, all residues achieved high specificity (0.933–0.971) and sensitivity (0.532–0.791). It should be noted that the ratio of NAD-/FAD-binding residues to nonbinding residues is about 1 to 16 in our dataset. This large difference might cause lots of false positives when predicted. That is the reason for high specificity and accuracy but low sensitivity in our prediction results. Hence, the positions of false positive residues in sequence were also investigated; 20% and 25% of false positive residues of NAD- and FAD-binding prediction occurred next to the true positive residues in sequence. It was shown that these residues are also located near the ligand in the coordinate space. If these residues were treated as true positive residues, our prediction results of NAD-binding yielded 71.55% sensitivity and 0.61 MCC at a 5% FPR threshold. Under the same conditions, FAD-binding-site predictions yielded 73.34% sensitivity and an MCC of 0.64. Compared with other prediction methods, ours did not use protein evolutionary information but only used protein structure and did not need to use equal number dataset for training but predicted whole-proteins through comparing structures of template database. Our results yielded excellent prediction performance when analyzing NAD-/FAD-binding residues and thus provide important details concerning the binding-site microenvironment. This approach, therefore, may be used to predict putative NAD-/FAD-binding proteins and the specific residues involved in the interaction.

## 4. Methods 

### 4.1. Overview

**Figure 8:**
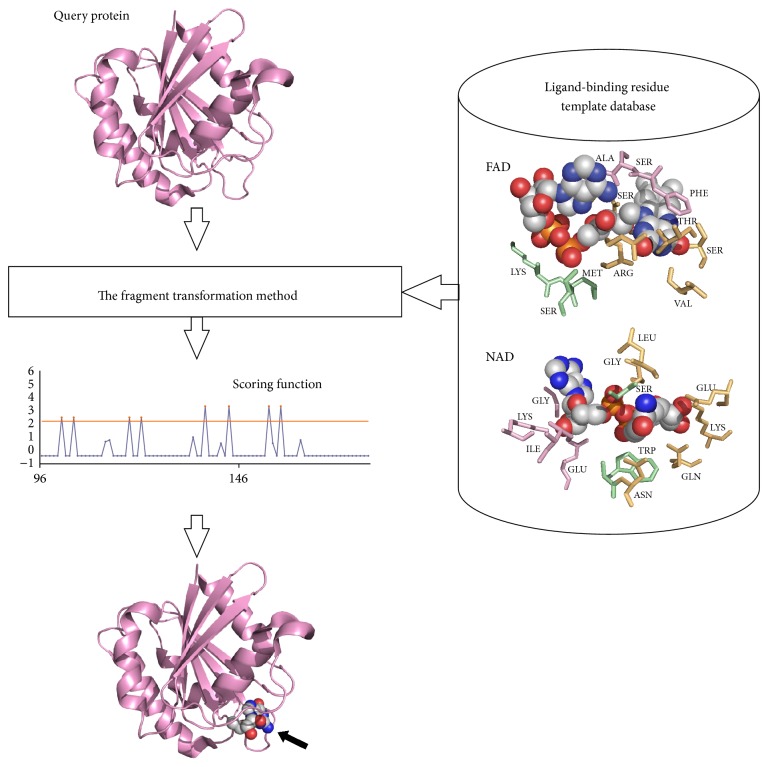
Schematic of the method for predicting NAD-/FAD-binding sites.

We extracted structures of proteins bound to NAD or FAD from PDB and constructed a database of NAD-/FAD-binding residue templates. Residues that were defined as binding residues by the ligand-binding database BioLiP [40] were included in the template. Query protein structures were then compared with each template in the database using a “leave-one-out” comparison method. The fragment transformation method [22] was used to align query and template structures. After comparing the local protein structure, each residue was assigned a score based on both protein sequence and structure. Sequence similarity was calculated using the BLOSUM62 substitution matrix [41], whereas structural similarity was calculated by measuring the root mean square deviation (RMSD) of the C*α* carbons from local structure alignments and using a secondary structure substitution matrix [22] according to the Dictionary of Secondary Structure of Proteins' (DSSP) definition of secondary structure [42]. Residues with an alignment score that exceeded a predetermined threshold were predicted to bind NAD/FAD. This method is illustrated in Figure 8.

### 4.2. NAD-/FAD-Binding Proteins and Binding Residue Templates

**Figure 9:**
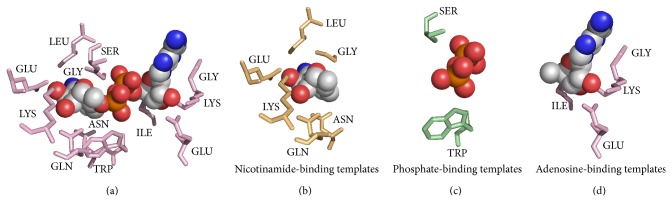
NAD-binding residue templates. (a) The entire NAD-binding template. (b) Nicotinamide-binding templates. (c) Phosphate-binding templates. (d) Adenosine-binding templates.

**Figure 10:**
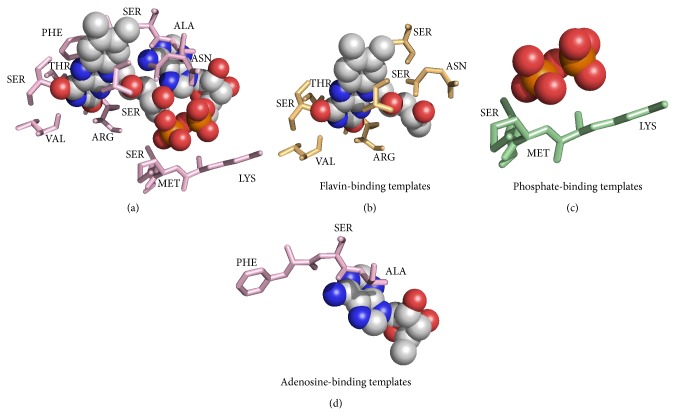
FAD-binding residue templates. (a) The entire FAD-binding template. (b) Flavin-binding templates. (c) Phosphate-binding templates. (d) Adenosine-binding templates.

We adopted the same datasets with previous research [10, 11]. All protein complexes were collected from PDB and had pairwise sequence identity <40% by using CD-HIT. Proteins chains that are not involved in NAD/FAD binding were excluded. Residues that were defined as binding or nonbinding residues by using the ligand-binding database BioLiP. The main dataset included 184 and 165 polypeptide chains for NAD and FAD, respectively. Because NAD is composed of a nicotinamide moiety, an adenosine moiety, and a phosphate moiety, binding residues were divided into three groups: nicotinamide binding, adenosine binding, and phosphate binding. FAD-binding sites similarly contain flavin-binding residues, adenosine-binding residues, and phosphate-binding residues. Groups of residues that contained more than or equal to two binding residues were considered a binding residue template (see Figures 9 and 10).

### 4.3. The Fragment Transformation Method

We used the fragment transformation method to align NAD-/FAD-binding residues. Each residue was treated as an individual unit and was used to align the query protein *S* with the binding template *T*. The structural unit consists of a triplet formed by the N–C_*α*_–C atoms within a given residue. *S* denotes the query protein of length *m*, and *T* denotes the template of *n* residues. The query protein *S* of length *m* and the template *T* of *n* residues can therefore be expressed in terms of triplets as *S* = {*σ*_1_, *σ*_2_,…, *σ*_*m*_} and *T* = {*τ*_1_, *τ*_2_,…, *τ*_*n*_}, where *σ*_*i*_ = (*p*_*N*_, *p*_*Cα*_, *p*_*C*_), *τ*_*j*_ = (*q*_*N*_, *q*_*Cα*_, *q*_*C*_), and *p* and *q* are PDB coordinates for each atom.

A matrix of dimensions *m* × *n* was then constructed for the residues of *S* and *T* as

(1) $$\begin{array}{c} M=\left|\begin{array}{cccc} M_{1,1} & M_{1,2} & \cdots & M_{1,n}\\ M_{2,1} & M_{2,2} & \cdots & M_{2,n}\\ \cdots & \cdots & \cdots & \cdots \\ M_{m,1} & M_{m,2} & \cdots & M_{m,n} \end{array}\right|, \end{array}$$

where the element *M*_*ij*_ is a rigid-body transformation matrix that transforms the triplet *σ*_*i*_ to *τ*_*j*_ (i.e., *M*_*ij*_ *σ*_*i*_ = *τ*_*j*_).

### 4.4. Performing Triplet Clustering

*D*_*kl*_^*ij*^ is the Cartesian distance between the target *τ*_*l*_ and the transformed triplet *M*_*ij*_ *σ*_*k*_, providing a measure of how similarly the triplet pairs (*σ*_*i*_, *τ*_*j*_) and (*σ*_*k*_, *τ*_*l*_) are oriented. This allows clustering of triplet fragments using the single-linkage algorithm [43] as follows. If for two triplet pairs, (*σ*_*i*_, *τ*_*j*_) and (*σ*_*k*_, *τ*_*l*_), *D*_*kl*_^*ij*^ < *D*_0_, *i* ≠ *k* and *j* ≠ *l*, then the triplets are clustered. Let *G*_1_ and *G*_2_ be two clusters, with the first containing (*σ*_*i*_, *τ*_*j*_) and (*σ*_*k*_, *τ*_*l*_) and the second containing (*σ*_*i*′_, *τ*_*j*′_) and (*σ*_*k*′_, *τ*_*l*′_). If *D*_*k*′*l*′_^*ij*^ < *D*_0_, then *G*_1_ and *G*_2_ are merged to form a new cluster *G*_3_, where *G*_3_ = *G*_1_ ∪ *G*_2_. These procedures are performed iteratively until no new clusters can be formed. For each final cluster *G*_*μ*_, we can obtain the transformation matrix *M*_*k*,*l*_^*μ*^ and aligned substructure pair *S*_*μ*_ = ⋃_*σ*_*k*_∈*G*_*μ*__ *σ*_*k*_ and *T*_*μ*_ = ⋃_*τ*_*l*_∈*G*_*μ*__ *τ*_*l*_, where *G*_*μ*_ has the minimum Cartesian distance when using *M*_*k*,*l*_^*μ*^.

### 4.5. Scoring Function

For each residue *i*, the binding score *C*_*i*_ is defined as

(2) $$\begin{array}{c} C_{i}=\underset{\sigma_{i}\in G_{\mu }}{\text{MAX}}\left(\varepsilon_{\mu }\times C_{\mu }^{R}\times C_{\mu }^{B}\times C_{\mu }^{D}\right), \end{array}$$

where *ε*_*μ*_ is the number of triplets of *S*_*μ*_ (i.e., the aligned residues of the query structure). The alignment scores *C*_*μ*_^*R*^, *C*_*μ*_^*B*^, and *C*_*μ*_^*D*^ are defined as

(3) $$\begin{array}{c} C_{\mu }^{R}=\frac{1}{1+\text{RMSD}\left(S_{\mu },T_{\mu }\right)},\\ C_{\mu }^{B}=\frac{\text{BLOSUM}\left(S_{\mu },T_{\mu }\right)}{\text{BLOSUM}\left(T_{\mu },T_{\mu }\right)}+1\\ C_{\mu }^{D}=\frac{\text{DSSP}\left(S_{\mu },T_{\mu }\right)}{\text{DSSP}\left(T_{\mu },T_{\mu }\right)}+1, \end{array}$$

where RMSD (*S*_*μ*_, *T*_*μ*_) is the RMSD of all *C*_*α*_ atoms between *S*_*μ*_ and *T*_*μ*_, BLOSUM (*S*_*μ*_, *T*_*μ*_) is the sequence alignment score between *S*_*μ*_ and *T*_*μ*_ calculated using the BLOSUM62 [41] substitution matrix, BLOSUM (*T*_*μ*_, *T*_*μ*_) is the maximum sequence alignment score of *T*_*μ*_, DSSP (*S*_*μ*_, *T*_*μ*_) represents the secondary structure alignment score based on a construction substitution matrix [22] using the definition of DSSP [42] between *S*_*μ*_ and *T*_*μ*_, and DSSP (*T*_*μ*_, *T*_*μ*_) is the maximum secondary structure alignment score of *T*_*μ*_. The value of RMSD (*S*_*μ*_, *T*_*μ*_) should be <3 Å.

For each residue *i*, we predict a geometric center Θ_*i*_^*ω*^ of the ligand by Θ_*i*_^*ω*^ = *M*_*k*,*l*_^*μ*^ ^−1^ *L*_*ω*_, where *L*_*ω*_ is the geometric center of the binding template type *ω* in template *T*. *ω* represents the three moieties of NAD/FAD: nicotinamide, adenosine, and phosphate for NAD; flavin, adenosine, and phosphate for FAD. The binding score *C*_*k*_ is added to *C*_*i*_ if the distance between Θ_*i*_^*ω*^ and Θ_*k*_^*ω*′^ is between 3 and 9 Å, and *ω* ≠ *ω*′. Finally, the normalized binding score *Z*_*i*_^*C*^ is calculated as

(4) $$\begin{array}{c} Z_{i}^{C}=\frac{C_{i}-\bar{C}}{SD_{C}}, \end{array}$$

where $\bar{C}$ and *SD*_*C*_ denote the mean and standard deviation, respectively, of the binding score *C*_*i*_.

### 4.6. Performance Assessment

The accuracy of predicting NAD-/FAD-binding sites wasdefined as the number of true positives and true negatives and was evaluated using a leave-one-out approach. Accuracy (ACC), the true positive rate (TPR), and the false positive rate (FPR) were calculated using true positive (TP), true negative (TN), false positive (FP), and false negative (FN) values as follows:

(5) $$\begin{array}{c} \text{ACC}=\frac{\text{TP}+\text{TN}}{\text{TP}+\text{TN}+\text{FP}+\text{FN}}\\ \text{TPR}=\text{Sensitivity}=\frac{\text{TP}}{\text{TP}+\text{FN}}\\ \text{FPR}=1-\text{Specificity}=\frac{\text{FP}}{\text{FP}+\text{TN}}. \end{array}$$

## Figures and Tables

**Table 1:** The performance of binding-site predictions at a 5% FPR threshold.

	Accuracy (%)	Sensitivity (%)	Specificity (%)	MCC
NAD	93.46	67.09	95.08	0.52
FAD	93.59	68.43	95.22	0.54

**Table 2:** The performance of binding-site predictions at a maximum MCC threshold.

	Accuracy (%)	Sensitivity (%)	Specificity (%)	MCC
NAD	95.34	57.88	97.64	0.57
FAD	94.33	64.13	96.27	0.55

**Table 3:** Comparison between the fragment transformation and SVM methods for predicting NAD-binding-site residues.

	Accuracy (%)	Sensitivity (%)	Specificity (%)	MCC
Random 1	87.46	86.45	88.48	0.75
Random 2	87.23	85.79	88.67	0.74
Random 3	87.38	85.65	89.11	0.75
Random 4	87.46	86.91	88.01	0.75
Random 5	87.38	86.25	88.51	0.75
Average	**87.38**	**86.21**	**88.56**	**0.75**

SVM [10]	87.25	86.13	88.37	0.75

**Table 4:** Comparison between the fragment transformation and SVM methods for predicting FAD-binding-site residues.

	Accuracy (%)	Sensitivity (%)	Specificity (%)	MCC
Random 1	87.38	85.68	89.08	0.75
Random 2	87.48	85.73	89.23	0.75
Random 3	87.35	85.55	89.15	0.75
Random 4	87.58	85.73	89.43	0.75
Random 5	87.44	85.73	89.15	0.75
Average	**87.45**	**85.68**	**89.21**	**0.75**

SVM [11]	82.86	83.36	82.36	0.66
